# Unilateral Auditory Neuropathy Caused by Cochlear Nerve Deficiency

**DOI:** 10.1155/2012/914986

**Published:** 2012-02-28

**Authors:** Cheng Liu, Xingkuan Bu, Feiyun Wu, Guangqian Xing

**Affiliations:** ^1^WHO Collaborating Centre for the Prevention of Deafness and Hearing Impairment, Nanjing Medical University, Nanjing 210029, China; ^2^Department of Radiology, First Affiliated Hospital of Nanjing Medical University, Nanjing 210029, China; ^3^Department of Otolaryngology, First Affiliated Hospital of Nanjing Medical University, Nanjing 210029, China

## Abstract

*Objective*. To explore possible corelationship between the cochlear nerve deficiency (CND) and unilateral auditory neuropathy (AN). *Methods*. From a database of 85 patients with unilateral profound sensorineural hearing loss, eight who presented with evoked otoacoustic emissions (EOAEs) or cochlear microphonic (CM) in the affected ear were diagnosed with unilateral AN. Audiological and radiological records in eight patients with unilateral AN were retrospectively reviewed. *Results*. Eight cases were diagnosed as having unilateral AN caused by CND. Seven had type “A” tympanogram with normal EOAE in both ears. The other patient had unilateral type “B” tympanogram and absent OAE but CM recorded, consistent with middle ear effusion in the affected ear. For all the ears involved in the study, auditory brainstem responses (ABRs) were either absent or responded to the maximum output and the neural responses from the cochlea were not revealed when viewed by means of the oblique sagittal MRI on the internal auditory canal. *Conclusion*. Cochlear nerve deficiency can be seen by electrophysiological evidence and may be a significant cause of unilateral AN. Inclined sagittal MRI of the internal auditory canal is recommended for the diagnosis of this disorder.

## 1. Introduction

Auditory neuropathy (AN) is a clinical syndrome characterized by the absence of, or the grossly abnormal, auditory brain stem response (ABR) in the presence of normal outer hair cell function as revealed by otoacoustic emission (OAE) and/or cochlear microphonic (CM) [1]. Most ANs show bilateral presentation accompanied by typical audiological features, but recent studies suggest that some AN cases involve only one ear [2–4]. This kind of patients could easily be missing because of the present EOAE during the newborn hearing screening. During the past year, eight unilateral AN cases were found in our hospital, and all of them were diagnosed as cochlear nerve deficiency (CND) on MRI. This findings support the routine use of MRI on unilateral AN.

## 2. Materials and Methods

### 2.1. Subjects

A retrospective review was undertaken at the First Affiliated Hospital of Nanjing Medical University. During the study period of August 2009 to August 2010, 85 patients (46 male and 39 female, between the ages of 1 and 26 years old) with unilateral profound sensorineural hearing loss were used to construct a detailed database including clinical manifestations, audiological characteristics, and radiological findings. Eight patients (9.4%) with electrophysiologic responses characteristic of AN were identified as unilateral cochlear nerve deficiency on MRI.

### 2.2. Evaluation of the Auditory System

 Puretone audiometry was performed using Madsen Orbiter 922 in a soundproof room. Hearing impairment was defined as the level of Puretone thresholds averaged at 0.5, 1, 2, and 4 kHz. Hearing loss of 26–40 dB was considered mild, 41–60 dB, moderate, 61–80 dB severe, and more than 80 dB profound. Tympanograms and acoustic reflex thresholds were measured using a Madsen Zodiac 901 Middle-Ear Analyzer. Tympanometry was obtained using a 226 Hz probe tone, with a sweep pressure start point of +200 daPa and an end point of −400 daPa. Ipsilateral and contralateral acoustic reflexes were assessed bilaterally with Puretone activator stimuli of 0.5, 1, 2, and 4 kHz. Transient EOAE (TEOAE) and distortion product EOAE (DPOAE) were recorded separately by Madsen CAPELLA systems. TEOAE was measured using click stimuli with a nonlinear mode of stimulus presentation and was considered “present” when the signal-to-noise ratio was ≥6 dB and the confidence ratio was 80%. DPOAE was recorded with a fixed ratio of f2/f1 = 1.2 and fixed levels of 65 dB SPL and 55 dB SPL. A DPOAE pass criterium was presented at a particular frequency region when the signal-to-noise ratio was ≥6 dB. The 100 *μ*sec click-evoked ABR was recorded using a band pass from 100 to 3000 Hz and in a 10 ms time window with 1024 averages. Click stimuli were rarefaction and condensation clicks presented at the rates of 11.1 per second. The ABR was undertaken using an ICS CHARTR EP system and was considered absent if there was no repeatable response to click stimulus at 97 dB nHL, the maximum intensity of the equipment, and normal if a response was present at lower than 30 dB nHL. CM was obtained simultaneously with ABR tests. The most direct method of separating the CM and ABR is to compare responses obtained with rarefaction polarity stimuli with those obtained with condensation stimuli as described by Berlin et al. [5]. CM follows the characteristics of the external stimulus; thus, the direction of the CM reverses with changes in polarity of the stimulus. ABR, however, may show only slight latency shifts with polarity changes and does not invert. All patients were quiet during the tests and children younger than six were put to sleep using chloral hydrate (50 mg/kg). 

### 2.3. MRI Technique

All patients were scanned using SIEMENS TrioTim 3.0T at a resolution of 0.5 mm × 0.5 mm × 0.5 mm. Direct and reconstructed sagittal oblique images of the contents of the internal auditory canal (cochlear, vestibular, and facial nerve) were obtained perpendicular to the long axis of the internal auditory canals.

## 3. Results

**Figure 1:**
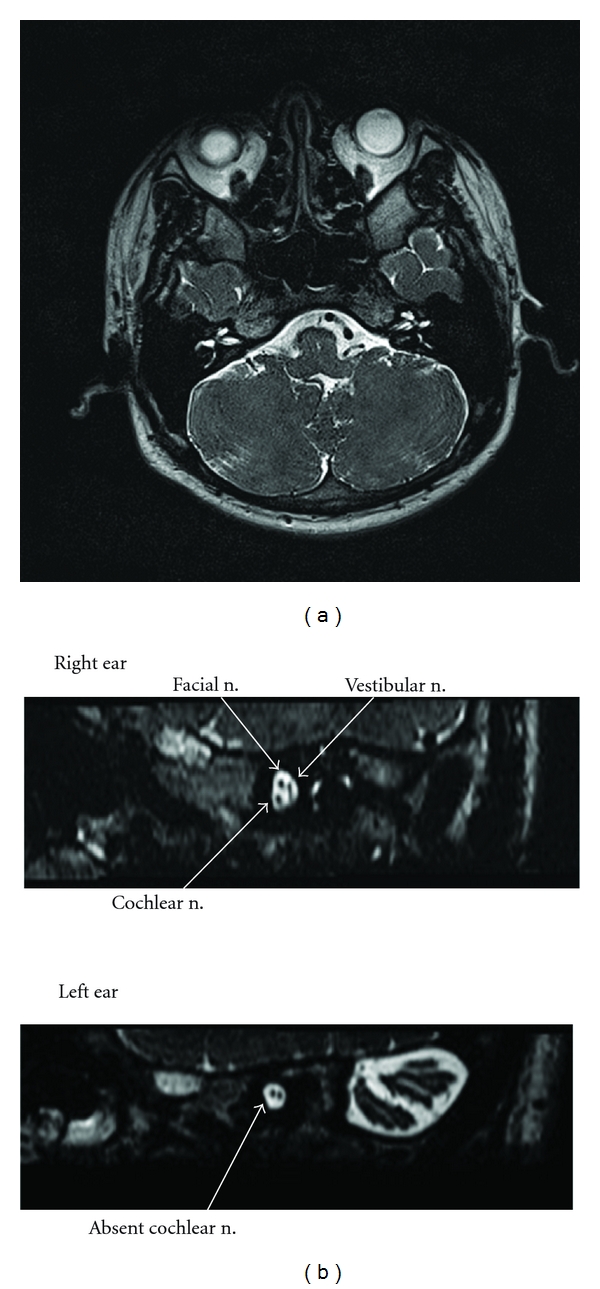
Axial 3-dimensional T2-weighted images through the internal auditory canal of case 1. The reconstructed sagittal oblique images were demonstrated. The right ear has a normal-size cochlear nerve as marked by arrow, while the left cochlear nerve is absent although facial nerve and vestibular nerve have their distinguishable locations.

**Figure 2:**
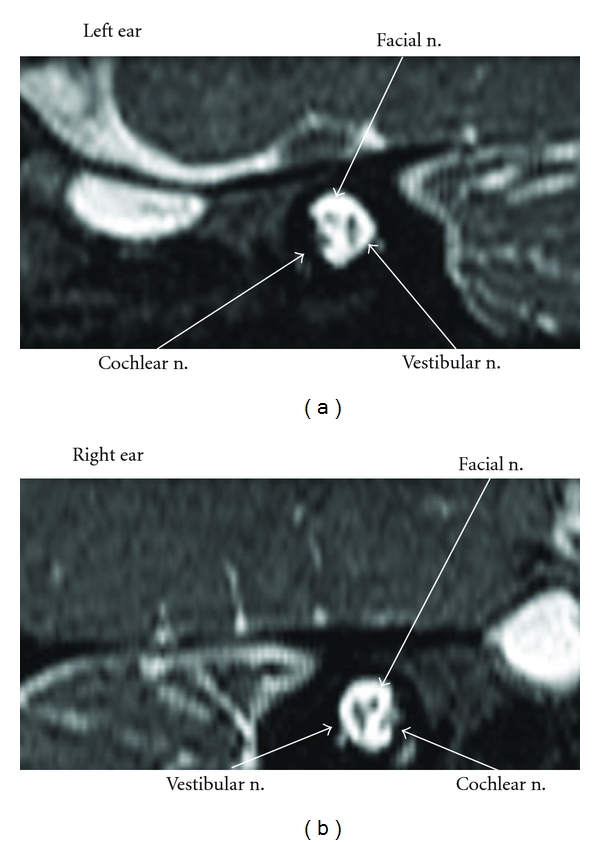
The images of normal cochlear nerve in a patient with unilateral profound sensorineural hearing loss.

85 patients with unilateral profound sensorineural hearing loss received hearing tests and an MRI examination. None of the patients had a history of prematurity, hypoxia, hyperbilirubinemia, exposure to ototoxic drugs, or other central nervous system disorders. No one had a family history of hearing loss. Vertigo, tinnitus, and other neuropathies were not reported. Speech and language were well developed. Physical examination revealed nothing remarkable except one case whose ear drum was retracted in the left ear. All patients but one had type “A” tympanogram in both ears. Ipsilateral acoustic reflexes were absent but contralateral was present with affected ear stimulation, contrary to the normal ear. One person had type “B” tympanogram with ipsilateral and contralateralacoustic reflexes were absent in the affected ear stimulation. In all cases ABRs were absent or responded to the maximum output in the worse ear, while being normal in the better one. EOAE and CM were present in seven patients. CM was only recorded in one case with absent EOAE indicating middle ear effusion. The neural responses from the cochlea of the eight patients were absent when viewed by means of the oblique sagittal MRI on the internal auditory canal.  Table 1 shows a summary of the findings for eight cases. There are five males and three females who ranged in age from 2 to 23 years.  Figure 1 shows the MRI of case 1 with a full complement of nerves in the right ear and an absent cochlear nerve in the left. Is the imaging of a patient with unilateral profound sensorineural hearing loss with normal cochlear nerve is shown in Figure 2.

## 4. Discussion

 ANs are thought to usually present bilaterally, but recent studies suggest that some AN cases involve only one ear [2–4], accounting for less than 10 percent of all ANs. The causes of unilateral AN are still unclear; however, cochlear nerve deficiency, either partially (hypoplasia) or completely (aplasia or agenesis), has recently been recognized as a significant cause of SNHL. A report of 148 cases of children with unilateral sensorineural hearing loss was provided by Laury et al. [6]. There were 11 children (7.4%) with normal EOAE in the affected ear. In these 11 patients, the current MRI technique was used with 10 children of which 8 were diagnosed as CND and 2 as neoplasm. Of a total of 271 imaged ears with SNHL in children, 49 (18%) demonstrated deficient (23 ears) or absent (26 ears) cochlear nerves [7]. We recently found eight unilateral ANs with MRI confirmation of CND in the past year, showing that unilateral AN may be caused by CND.

 A typical AN characteristic is a speech discrimination score out of sync with the level of hearing loss. Recently auditory neuropathy spectrum disorder has taken the place of AN to describe this kind of disease [8–10]. Hearing thresholds for Puretone detection can range from normal to profound levels. Other hearing features include type “A” tympanogram with missing acoustic reflex, normal EOAE and/or CM, and absent or grossly abnormal ABR. In our eight cases, clinical manifestations of AN were not revealed as the profound deafness involved only one ear. If complete audiological examinations were not used, these patients might be misdiagnosed indiscriminately as unilateral SNHL or sudden hearing loss. Puretone audiometry and CT scans were performed many times during the most recent ten years in case 1; the accurate diagnosis, however, was not made until comprehensive hearing assessments and MRI were conducted. Sometimes, other factors causing hearing loss may make us ignore exploring the true etiology for it. For example, traumatic deafness had already been diagnosed in case 3 because of the trauma history and case 4 had suffered OME in the affected ear, which covered the real etiology. It is conceived that patients with severe and profound unilateral SNHL had more abnormalities than ones with mild and moderate, and children with unilateral moderate, severe, or profound SNHL had more inner ear abnormalities than those with bilateral loss [7]. Buchman et al. also suggested that unilateral AN characteristics with profound hearing loss should highly suggest a diagnosis of CND [4]. All the eight subjects we documented had unilateral profound SNHL and they received the integral audiological and radiological imaging examinations starting with their first visit to our hospital. After analysis of the results, we were able to make the diagnosis accurately and provided the patients with the actual etiology of their hearing impairment.

 Buchman et al. reported that nine (13 ears) of 51 children with ABR characteristics of AN had been identified as having small (*N* = 2) or absent (*N* = 7) cochlear nerves on MRI [4]. Of these 13 ears, 9 had normal IAC size and morphology and only 4 ears had small IAC. The internal auditory canal and inner ear morphologic characteristics of 14 children (5 bilateral, 9 unilateral) with CND were evaluated by means of high-resolution MRI [11]. The study found that most CND had normal IAC morphology, while there was a cochlear nerve in the small IAC. Thus, the IAC morphology is an unreliable marker of CN integrity. Based on the findings, high-resolution MRI, rather than CT imaging, should be performed in cases of pediatric hearing loss, especially in those with profound hearing loss. In our point of view, the oblique sagittal MRI of internal auditory canal is very helpful in identifying CND, and both the direct and reconstructed images had equal diagnostic values Because of the limits of MRI, the imaging of cochlear nerve in some patients was too small to be detected, which made judgment difficult [12]. The determination of CND required detailed hearing tests, physical examination, and imaging information. Although there was no imaging of cochlear nerve on the MRI in the affected ear in case 2, residual hearing in his right ear led us to the diagnosis of cochlear nerve hypoplasia (small) rather than absence of nerve as indicated in other 7 cases.

 The temporal bone histopathology of unilateral profound SNHL was studied by Nelson and Hinojosa (2001) [13], in which the separation of the inner ear and cochlear nerve development was disclosed as demonstrated by two cases with unilateral cochlear nerve deficiency and normal organ of Corti structure and hair cells. The mechanism(s) responsible for CND remains speculative but may be caused by congenital dysplasia or acquired degeneration. Salvinelli et al. reported a 12-year-old female presented with unilateral profound SNHL and normal EAOE suffering an episode of parotitis two weeks before [14]. The author suspected that the mumps virus violated the cochlear nerve or inner hair cells instead of outer hair cells. Our case 2 had a history of mumps before his awareness of hearing loss, and we presumed that it caused the cochlear nerve degeneration and the resultant absence on MRI imaging. The results of the remaining 7 subjects were probably due to congenital dysplasia since there was no definite etiological evidence.

In conclusion, cochlear nerve deficiency can present with electrophysiological evidence and may be a significant cause of unilateral AN. Inclined sagittal MRI rather than CT scanning plays a very important role in the precise diagnosis of this disorder. As contralateral hearing loss in a progressive manner in a patient with unilateral CND had recently been reported [4], the continued audiological observation of the unaffected ear is also needed.

## Figures and Tables

**Table 1:** Audiological characteristics and radiological findings in patients with CND.

Subject	Age (years)	Gender	Affected ear	PTA	Tym type	EOAE	ABR	CM	Risk factors for SNHL	Other Ear diseases	CN
1	23	M	Left	NR	A	Present	NR	Present	None	None	Absent
2	14	M	Right	97 dB HL	A	Present	Present*	Present	parotitis	None	Small
3	4	M	Right	NA	A	Present	NR	Present	None	None	Absent
4	7	F	Left	NR	B	Absent	NR	Present	None	OME	Absent
5	10	F	Left	NR	A	Present	NR	Present	None	None	Absent
6	2	F	Left	NA	A	Present	NR	Present	None	None	Absent
7	3	M	Right	NA	A	Present	NR	Present	None	None	Absent
8	5	M	Left	NR	A	Present	NR	Present	None	None	Absent

PTA: pure tone average, Tym: tympanogram, EOAE: evoked otoacoustic emissions, ABR: auditory brainstem response, SNHL: sensorineural hearing loss, CN: cochlear nerve, NR: no response, and NA: not available. *ABR threshold was 97 dB nHL; OME: otitis media with effusion.
